# Work-related musculoskeletal symptoms among Iranian nurses and their relationship with fatigue: a cross-sectional study

**DOI:** 10.1186/s12891-021-04510-3

**Published:** 2021-07-19

**Authors:** Elahe Hosseini, Hadi Daneshmandi, Azadeh Bashiri, Roxana Sharifian

**Affiliations:** 1 Health Human, Resources Research Center, Department of Health Information Technology, School of Management and Medical Informatics, Shiraz University of Medical Sciences, Shiraz, Iran; 2 Research Center for Health Sciences, Institute of Health, Shiraz University of Medical Sciences, Shiraz, Iran; 3 Health Human Resources Research Center, Clinical Education Research Center, Department of Health Information Technology, School of Management and Medical Information Sciences, Shiraz University of Medical Sciences, Shiraz, Iran; 4 Health Human Resources Research Center, Department of Health Information Technology, School of Management and Medical Information Sciences, Shiraz University of Medical Sciences, Shiraz, Iran

**Keywords:** Fatigue, Musculoskeletal symptoms, Hospital nurses, Risk factor

## Abstract

**Background:**

The present study aimed to determine the prevalence of work-related musculoskeletal symptoms (WMSs), identify potential factors associated with WMSs, and determine the association between WMSs and fatigue among nurses.

**Methods:**

This cross-sectional study was carried out among 500 Iranian nurses. Data was gathered by the 1) Persian version of the Nordic musculoskeletal questionnaire (P-NMQ) to examine WMSs, and 2) Persian version of the Multidimensional Assessment of Fatigue (P-MAF) Scale to evaluate fatigue among the study population. Then, data was analyzed by SPSS version 21 using the χ2 test, multiple logistic regression for detection of potential factors associated with WMSs, and multiple linear regression for detection of potential factors associated with fatigue.

**Results:**

Ankles/feet, lower back, knees, and shoulders had the highest prevalence of WMSs among nurses within the last 12 months prior to the study. Independent variables including age, job tenure, gender, smoking, shift work, and type of employment were significantly associated with WMSs in different body regions with odds ratios (ORs) ranging from 1.635–2.835. Moreover, WMSs in some body regions were associated with subscales of fatigue and total fatigue.

**Conclusions:**

Ergonomic and organizational interventions for fitting the job to the nurses considering demographic/occupational characteristics are highly essential to improve musculoskeletal system health and relieve fatigue.

## Introduction

Work-related musculoskeletal symptoms (WMSs) are common painful disorders affecting the body structure, i.e. joints, tendons, muscles, and nerves. These symptoms can occur in the back, upper, and lower limbs [1, 2]. Work-related musculoskeletal disorders (WMSDs) are an important concern for any organization’s human resources due to costs, illness, cure, productivity, legal, and injury issues [3]. Many researchers consider WMSDs as a growing problem in the world [4–6]. WMSDs account for 29% of all US workplace injuries [1, 2]. In the United States and Canada, upper limb disorders and low back pain play a significant role in work-related injuries [7]. The prevention of WMSDs in the workplace requires the identification of the most important individual and occupational risk factors related to symptoms and exclude these causal factors from the workplaces [8].

WMSDs are repetitive strain injuries, which are known as the most common work-related health problems and causes of fatigue. These symptoms might become apparent after days, months, or even years of exposure to workplace risk factors [9, 10]. Previous studies have suggested that WMSDs might be caused by the development of fatigue in musculoskeletal structures [11].

Nurses in hospitals usually work in poor ergonomic working conditions for a long period of time, which can lead to an increase in MSDs, fatigue, and loss of efficiency [12].

Previous studies have revealed that WMSDs are a common occurrence among nurses [13]. The findings of a study by Tinubu et al. revealed that the 12-month period and point prevalence rate of WMSDs in at least one body region of Nigerian nurses were 78% and 66.1%, respectively. They also stated that WMSDs occurred mainly in the low back (44.1%), neck (28.0%), and knees (22.4%) [14]. Along the same lines, Chiwaridzo et al. demonstrated that the prevalence rate of MSD symptoms was 82.1% in studied nurses over the last 12 months prior to their study, and low back pain was the most common WMSD reported (67.9%) [13]. Researchers have also reported the prevalence of fatigue in specific populations as ranging from 7 to 45% [15, 16]. Nurses can be affected both mentally and physically; therefore, it is imperative for managers to be alert to the risks that fatigue may impose on nursing staff and the organization [15]. One essential aspect of nursing health and safety is work-related fatigue, which is known as the main source of harmful effects to the quality, satisfaction, and safety of patients and nurses [17].

WMSDs and fatigue are important issues that are sometimes neglected in healthcare workers such as nurses. In the hospital, nurses may encounter diverse musculoskeletal disorder risk factors. For example, long hours with a high mental workload were observed among hospital nurses. Awkward postures, highly dynamic repetitive activities, and patient handling are also very common among nurses. Moreover, nurses work long hours and have only a short rest period, which might cause fatigue [15]. In these situations, a high WMS occurrence rate is expected among hospital nurses. Assessing WMSs and fatigue and identifying potential associated factors among hospital nurses are important issues to better management, the prevention of WMSD symptoms and workforce disability, the promotion of job satisfaction, efficiency, effectiveness, and improved service delivery to patients. The present study was, therefore, undertaken among nurses with the following objectives:

- Determine the prevalence of WMSs and identify potential associated factors in nurses
- Determine the association between WMSs in different body regions and fatigue among nurses

## Methods

### Participants

In this cross-sectional study conducted in 2020, 500 Iranian nurses with at least one year of work experience from Shiraz teaching hospitals took part (participation rate: 89.28%). The participants were selected using a two-stage sampling method. First, the required number of participants was determined using the proportion method in each hospital; then samples from each hospital were selected by simple random sampling using a random number table. Employees with underlying diseases or having had accidents affecting their musculoskeletal system were excluded from the study. All participants had a bachelor's degree or higher.

The study was explained orally to potential subjects and all pertinent information such as purpose, procedures, risks, benefits, and alternatives to participation was provided. All subjects were allowed an ample opportunity to ask questions. Those nurses willing to cooperate in this study signed a written informed consent form and were enrolled. This study was approved by the local Ethics Committee of Shiraz University of Medical Sciences and conducted according to the Helsinki Declaration and its later amendments [18].

The participants completed the questionnaires described below through self-reporting at their workplace during their work shift.

In this study, data was collected using questionnaires as follows:

### Demographic and occupational questionnaire

This questionnaire included questions on variables such as age, height (cm), weight (kg), job tenure (year), working hours per day, gender (male/female), marital status (single/married), number of children, smoking (yes/no), shift work (yes/no), and type of employment (formal = *permanent employment*/contractor = *transient employment based on a contract*).

### Persian version of the Nordic musculoskeletal questionnaire (P-NMQ)

This questionnaire was used to examine reported musculoskeletal symptoms in different body regions among the study population [19]. In this study, symptoms were reported for the last 12 months prior to the study, one week before, and at present. Each participant filled out the questionnaire in his/her workplace. The psychometric properties of the Persian version of NMQ were examined by Choobineh et al. [20].

### Persian version of the multidimensional assessment of fatigue (P-MAF) Scale

The Multidimensional Assessment of Fatigue (MAF) scale, developed by Belza et al., contains 16 items that assess various aspects of fatigue. This tool is a self-administered questionnaire measuring four dimensions of fatigue, including degree and severity, amount of distress it causes, its timing, and the degree to which fatigue interferes with daily living activities. Items 1–14 are rated on a numerical scale [1–10], and items 15 and 16 (timing items) are rated on a categorical scale [1–4]. Finally, a Global Fatigue Index (GFI) was calculated. For GFI, the score range is 1–50 (1 = *no fatigue;* 50 = *severe fatigue*). To calculate the GFI, the rated score of item 15 [1–4] should first be converted into a 10-point scale by multiplying it by 2.5. Then, GFI is calculated by the following formula: GFI score = Summation of rated scores of items “1–3” + Average of rated scores of items “4–14” + New score of item “15”. Item 16 is not included in the GFI [21].

Participants in the current study were asked to reflect upon their experiences with respect to fatigue within the past week. The psychometric properties of the P-MAF scale have been examined by Daneshmandi et al. [12].

After collecting the data from each subject, the questionnaires were checked by the researchers. If important data had not been inserted, the participant was asked to present any questions or ambiguities, and after receiving an explanation, enter the missing information.

### Statistical analysis

In this study, data was analyzed using IBM SPSS version 21, χ^2^ test, multiple logistic regression, and multiple linear regression, as discussed below.

a) χ2 test, and multiple logistic regression

Multiple logistic regression (Forward Wald) was used to determine factors associated with WMSs in different body regions. In the first step, the association between variables, such as age, body mass index (BMI), job tenure, working h/day, gender, marital status, number of children, smoking, and shift working with musculoskeletal symptoms in different body regions were surveyed by the χ^2^ test with a significance level of *p* ≤ 0.25 [22]. For this aim, the quantitative variables were divided into two categories (age ≤ 35 years and age > 35 years, BMI ≤ 24.9 and BMI > 25, number of children = 0 and number of children ≥ 1, job tenure ≤ 10 years and job tenure > 10 years, and working h/day ≤ 8 h and working h/day > 8 h). Subsequently, all variables that had an association at *p* ≤ 0.25 in the binary analysis were included in the multiple logistics regression to control the effects of other variables on the association between WMSs and fatigue.

b) Multiple linear regression

To determine the association between WMS and fatigue among nurses, multiple linear regression analyses with forward selection were conducted using fatigue subscales as dependent variables and WMSs in different body regions, age, BMI, job tenure, working hours per day, working hours per week, gender, marital status, smoking, shift work, and type of employment as independent variables. A *p-*value < 0.05 was considered statistically significant.

## Results

Table 1 shows the personal and occupational details of the nurses who participated in the study. As shown, most of the subjects were female (77.8%).

**Table 1:** Some personal and occupational details of the participants (N = 500)

*Quantitative variables*	*Mean* ± *SD*	*Min—Max*
Age (years)	31.78 ± 6.89	24–63
Height (cm)	166.00 ± 7.97	142–189
Weight (kg)	67.10 ± 12.16	42–136
Body mass index	24.29 ± 3.68	16.30–40.57
Job tenure (years)	7.55 ± 6.43	1–28
Working hours per day	8.77 ± 1.37	6.67–13.33
Working hours per week	52.65 ± 8.27	40–80
*Qualitative variables*	***No. (%)***	
Gender		
Male	111 (22.2)	
Female	389 (77.8)	
Marital status		
Single	218 (43.6)	
Married	282 (56.4)	
Smoking		
Yes	35 (7)	
No	465 (93)	
Shift work		
Yes	455 (91)	
No	45 (9)	
Type of employment		
Formal	184 (36.8)	
Contractor	316 (63.2)	

Note: Type of employment: *Formal* Permanent employment, *Contractor* Transient employment based on a contract

The main findings are presented in two sections, as follows:

### WMSs and potential risk factors

The prevalence of WMSs in different body regions and their associated potential risk factors among the study population are presented in this section.

#### Prevalence of WMSs

**Fig. 1:**
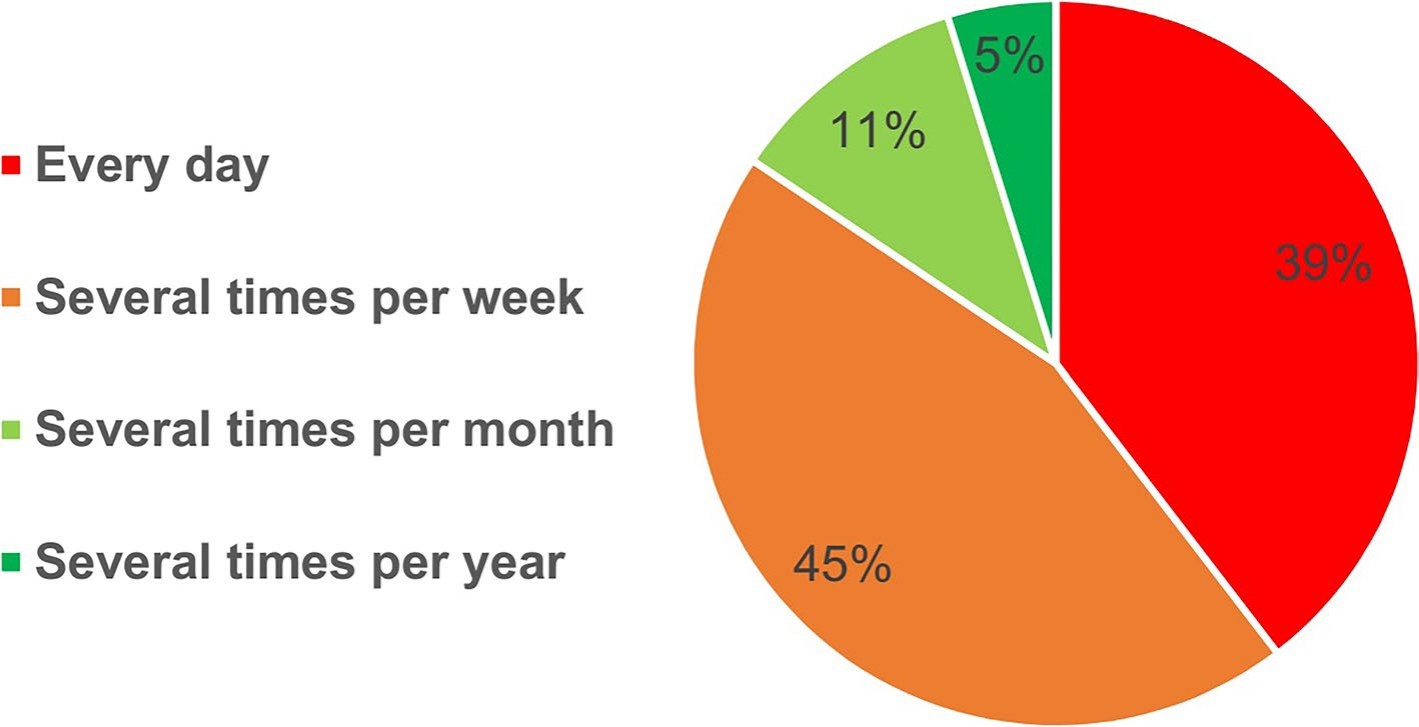
Frequency occurrence of WMSs during the past 12 months

**Fig. 2:**
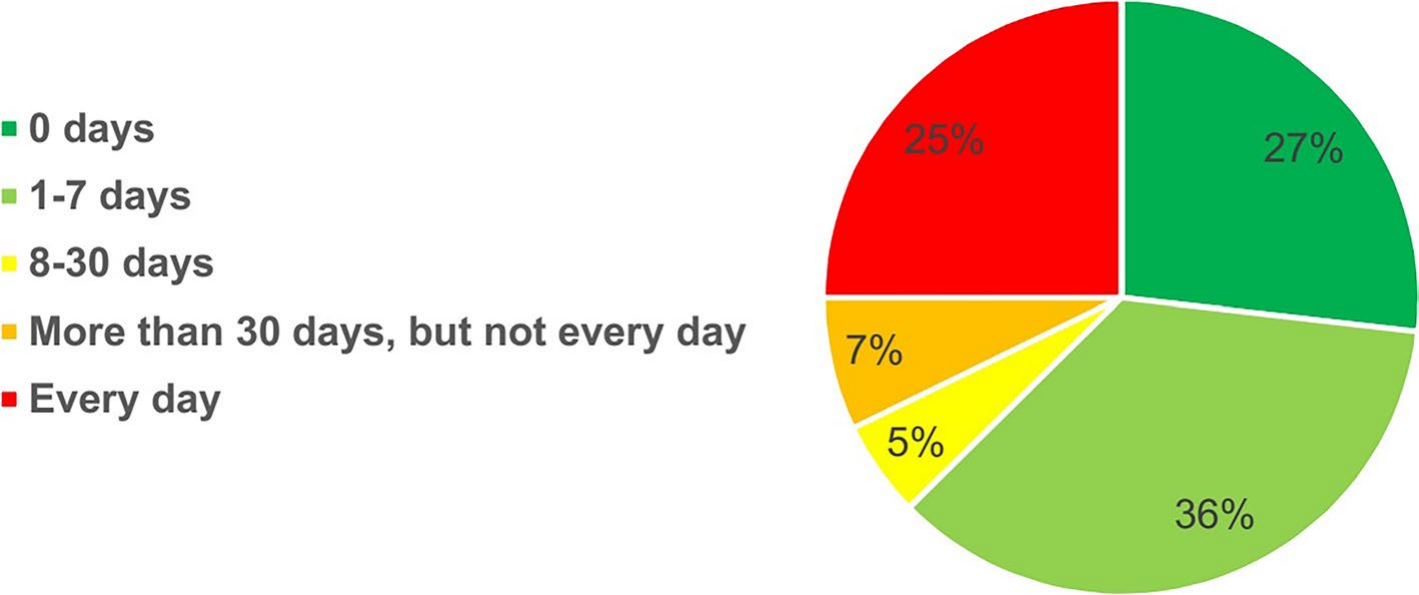
Duration of WMSs during the past 12 months

Figures 1 and 2 present the frequency of occurrence and time length of WMSs during the last 12 months prior to the study, respectively. As shown in Fig. 1, WMSs occurred ‘every day’ (39%) or ‘several times per week’ (45%) in 84% of studied nurses. Additionally, 37% of the participants reported a duration of WMSs of more than ‘7 days’ during the reported 12 months.

Table 2 shows the prevalence rate of the reported WMSs in different body regions among the nurses during the reported 12 months, the past week, and at present.

**Table 2:** The prevalence rate of the reported WMSs in different body regions among the studied nurses (N = 500)

Body region	During the last 12 months No. (%)	During the past week No. (%)	At present No. (%)
Neck	305 (39)	286 (57.2)	241 (48.2)
Shoulders	312 (62.4)	271 (54.2)	239 (47.8)
Elbows	58 (11.6)	4 (8.2)	38 (7.6)
Wrists/Hands	287 (57.4)	260 (52)	205 (41)
Upper back	314 (62.8)	288 (57.6)	244 (48.8)
Lower back	401 (80.2)	392 (78.4)	322 (64.4)
Thighs	140 (28)	117 (23.4)	98 (19.6)
Knees	316 (63.2)	285 (57)	244 (48.8)
Ankles/Feet	409 (81.8)	380 (76)	350 (70)
WMSs in at least one body region	488 (97.6)	487 (97.4)	459 (91.8)

#### Potential risk factors for WMSs

Table 3 shows the association between potential risk factors and WMSs in different body regions of participants in the reported 12 months using multiple logistic regression. As shown, the independent variables of age, job tenure, gender, smoking, shift work, and type of employment were significantly associated with WMSs in nurses’ different body regions.

**Table 3:** Modeling on the association between potential risk factors and WMSs in different body regions of participants in the last 12 months using multiple logistic regression (N = 500)

Body region	Association between potential risk factors and WMSs			
	**Potential risk factor**	**OR**	**95% CI**	p^*^
**Neck**	Type of employment			
	Contract employees: *Reference*	1.635	1.138–2.349	0.008
	Formal employees			
**Shoulders**	Smoking			
	No: *Reference*	2.835	1.389–5.787	0.004
	Yes			
	Shift work			
	No: *Reference*	2.388	1.120–5.089	0.024
	Yes			
**Elbows**	Age (years)			
	≤ 35: *Reference*	1.777	1.006–3.167	0.047
	> 35			
**Wrists/Hands**	-	-	-	-
**Upper back**	Age (years)			
	≤ 35: *Reference*	2.119	1.35–3.324	0.001
	> 35			
**Lower back**	Job tenure (years)			
	≤ 10: *Reference*	1.782	1.054–3.013	0.031
	> 10			
**Thighs**	-	-	-	-
**Knees**	Gender			
	Male: *Reference*	1.772	1.152–2.727	0.009
	Female			
	Shift work			
	No: *Reference*	2.649	1.200–5.850	0.016
	Yes			
**Ankles/Feet**	Gender			
	Male: *Reference*	2.266	1.380–3.718	0.001
	Female			
**WMSs at least in one body region**	Age (years)			
	≤ 35: *Reference*	1.134	1.024–2.843	0.003
	> 35			
	Job tenure (years)			
	≤ 10 years: *Reference*	1.604	1.078–2.98	0.034
	> 10 years			

^*^p < 0.05

Note: *p* = *p*-value, Multiple logistic regression

### WMSs and fatigue

Associations between total fatigue and the fatigue subscales and WMSs in different body regions of the participants are presented in this section.

The mean ± standard deviation and minimum and maximum scores of P-MAF subscales are presented in Table 4. As shown, the highest and lowest mean scores of these subscales were related to “degree and severity” and “timing of fatigue”, respectively.

**Table 4:** Descriptive features of P-MAF subscales (N = 500)

P-MAF subscale	Mean ± SD	Min—Max
Degree and severity	7.64 ± 1.79	1–10
Distress that it causes	7.21 ± 2.29	1–10
Degree of interference with activities of daily living	5.38 ± 2.00	1–10
Timing of fatigue	4.60 ± 2.02	3–10
Total fatigue/Global Fatigue Index	32.46 ± 5.59	14–48

Table 5 shows the association between WMSs in different body regions of the participants and P-MAF subscales.

**Table 5:** Associations between WMSs in different body regions of the participants and total fatigue and the fatigue subscales (N = 500)

**P-MAF subscale**	**Independent variable**	**Beta**	**95% CI**	***p***^a^
**Degree and severity**	Age	1.89	1.31–2.71	< 0.001
	Female gender	2.16	1.55–2.78	< 0.001
	Shoulders symptoms	1.90	1.05–2.37	< 0.001
	Upper back symptoms	2.03	1.21–2.65	< 0.001
	Lower back symptoms	2.11	1.31–2.75	0.009
**Distress that it causes**	Age	1.78	1.14–2.17	0.009
	Female gender	1.97	1.17–2.33	< 0.001
	Neck symptoms	2.14	1.11–2.66	0.001
	Shoulders symptoms	2.01	1.23–2.41	0.029
	Lower back symptoms	2.09	1.27–2.77	0.027
**Degree of interference with activities of daily living**	Age	2.12	1.35–2.83	0.006
	Female gender	2.18	1.26–2.79	< 0.001
	Marital status (married)	1.54	1.05–1.96	< 0.001
	Elbows symptoms	2.12	1.33–2.71	0.004
	Upper back symptoms	2.31	1.44–2.95	0.011
**Timing of fatigue**	Age	1.76	1.13–2.27	0.020
	Female gender	2.16	1.19–2.87	< 0.001
	Shoulders symptoms	2.19	1.27–2.96	< 0.001
	Lower back symptoms	3.12	1.77–3.88	0.005
	Thighs symptoms	1.49	1.05–1.97	0.038
**Total fatigue/Global Fatigue Index**	Age	2.34	1.52–2.98	0.025
	Female gender	2.45	1.34–3.01	< 0.001
	Neck symptoms	2.66	1.45–3.25	0.046
	Shoulders symptoms	2.56	1.37–3.17	< 0.001
	Upper back symptoms	2.31	1.41–2.98	0.013
	Lower back symptoms	2.77	1.50–3.41	< 0.001

^a^ Multiple linear regression analysis of P-MAF subscales and WMSs during the prior week adjusted for age, BMI, job tenure, working hours per day, working hours per week, gender, marital status, smoking, shift work, and type of employment. Only the statistically significant correlations are presented

Note: *p p*-value, Multiple linear regression (r^2^ = 0.49, *p* = 0.03)

## Discussion

This study aimed to investigate WMSs among Iranian nurses and their relationship with fatigue.

### WMSs and potential risk factors

#### Prevalence of WMSs

Based on the findings, WMSs occurred ‘every day’ or ‘several times per week’ in 84% of the studied nurses. Additionally, 37% of the participants reported a duration of WMSs of more than ‘7 days’ during the 12 months prior to the study.

The highest prevalence rates of WMSs during the reported 12 months, the past week, and at present were related to the ankles/feet, lower back, and ankles/feet, respectively. In contrast, the lowest prevalence of WMSs in the three time periods was related to participants’ elbows; this result is in line with those of Choobineh et al. [23].

The prevalence of WMSs during the 12 months prior to the study in some body regions of the studied population, including shoulders, wrists/hands, upper back, lower back, knees, and ankles/feet, was higher than that reported among other Iranian working groups, comprising healthcare providers [23], hospital attendants [24], workers in orthotic and prosthetic workshops [25], office workers [23, 26–28], assembly line workers [29], petrochemical industry workers [30], manufacturing industries workers [23], and agricultural workers [31].

The results regarding the prevalence of neck symptoms during the reported 12 months were similar to the results of Harcombe et al. [32]. However, the prevalence of lower back symptoms reported in the current study was higher than that of other studies among New Zealand [33] and Iranian [34] hospital nurses. In addition, the prevalence of WMSs at least in one body region of the study population was higher than the findings of Harcombe et al. [32]. The high prevalence of WMSs in the current study may be attributed to the high number of female nurses compared to males (77.8% female *vs.* 22.2% male). Some studies have shown that certain WMSs are more prevalent in women than men due to anatomical and hormonal differences [35].

### Potential risk factors for WMSs

Age was a potential risk factor for elbows and upper back symptoms. Based on the results, as age increases, the prevalence of WMSs also increases, which is in line with the results of other studies [36]. A positive association was observed between job tenure and lower back symptoms in the study population. Individuals with more than ten years of work experience were more susceptible to lower back symptoms than those with fewer years of work experience. These findings are similar to the results of other studies [30]. One significant cause of this claim is sarcopenia, a phenomenon that occurs at older ages (or high work experience) and is associated with degenerative loss of skeletal muscle mass, quality, and strength [37]. Under these circumstances, it is expected that the body’s repair process will take longer.

Gender was a potentially significant risk factor for knees and ankles/feet symptoms, such that female nurses were more likely to develop these symptoms than their male colleagues. A positive association was also found between shift work and shoulders and knees symptoms, which was in line with other studies [29, 37]. Based on interviews with formal nurses, it was found that they have higher ranking organizational positions. Therefore, this working group experiences a higher mental workload and stress in management issues and decision-making, factors which can play a significant role in the development of WMSs in the neck.

Analysis showed a significant association between type of employment and neck symptoms, which is in agreement with previous surveys [30]. The results further showed that smoking was a potential risk factor for the development of shoulder symptoms in the studied population. Other studies revealed similar results about the effect of smoking on developing WMSs [38, 39]. In this regard, it is specified that smokers tend to heal in the musculoskeletal system more slowly due to decreased oxygen in the bloodstream [40].

In general, age and job tenure were associated with WMSs in at least one body region. This means that as the age and job tenure increase, the chances of WMSs in nurses’ body regions increase as well. In this context, many studies have revealed a relationship between age and job tenure with WMSs [28, 30, 33, 38, 41, 42].

### WMSs and fatigue

The current findings showed that the mean ± standard deviation of total fatigue was 32.46 ± 5.59, representing a greater than average fatigue.

The findings also indicated that neck symptoms were associated with the ‘distress that it causes’ subscale and ‘total fatigue’. Shoulders and lower back symptoms were linked to ‘degree and severity’, ‘distress that it causes’, ‘timing of fatigue’ subscale, and ‘total fatigue’. Elbow symptoms were associated with the subscale ‘degree of interference with activities of daily living’. Upper back symptoms were linked to ‘degree and severity’, ‘degree of interference with activities of daily living’, and ‘total fatigue’. Thigh symptoms were associated with ‘timing of fatigue’ subscale. In this context, previous studies have revealed that WMSs were associated with various dimensions of fatigue [28, 43, 44]. In addition, the current findings are in accordance with the results of Sirge et al. [45] and Chavalitsakulchai et al. [46].

It is necessary to mention that based on other studies, fatigue and WMS have an interactive effect. Some studies have found that the subscales of fatigue, such as ‘degree and severity’, ‘distress that it causes’, ‘degree of interference with activities of daily living’, and ‘timing of fatigue’, can influence the development of WMSs [28, 47]. Fatigue causes a reduction in performance due to a period of excessive activity followed by inadequate recovery time. Muscle fatigue is accompanied by a buildup of lactic acid in the working muscles. In return, individuals move more slowly when fatigued, so simple tasks can take longer, thus increasing the duration of exposure to other risk factors of WMSs [40].

The findings also demonstrated that age and gender were associated with all fatigue subscales and total fatigue. Some previous studies have also revealed that age could contribute to the development of fatigue in nurses [48, 49]. The current findings regarding the association between gender and fatigue were similar to those of the Thompson study [50].

### Limitations of the study

Given the cross-sectional nature of this study and data collection by self-report, the findings should be interpreted cautiously. As another limitation in the current study, data related to other factors affecting the development of WMSs in nurses (e.g., second job, patient acuity, and organizational issues including patient-to-nurse ratio and management style) was not considered. Moreover, this study was performed among hospital nurses in Shiraz. Therefore, the results might not be generalizable to other hospital nurses and working groups.

## Conclusions

Ankles/feet, lower back, knees, and shoulders had a higher prevalence of WMSs in nurses in the 12 months prior to the study. In addition, independent variables, including age, job tenure, gender, smoking, shift work, and type of employment, were significantly associated with WMSs in different body regions of nurses. Moreover, WMSs in some body regions of nurses were associated with the subscales of fatigue and total fatigue.

Ergonomic and organizational interventions for fitting the job to the nurses considering demographic/occupational characteristics are essential to improving musculoskeletal system health, especially in the ankles/feet, lower back, knees, and shoulders regions, and to relieving fatigue.

## Data Availability

The datasets used and/or analysed during the current study are available from the corresponding author on reasonable request.

## Abbreviations

WMSs: Work-related musculoskeletal symptoms
WMSDs: Work-related musculoskeletal disorders
P-NMQ: Persian version of the Nordic musculoskeletal questionnaire
P-MAF: Persian version of the multidimensional assessment of fatigue Scale
OR: Odds ratio
